# Willingness to pay for rapid diagnostic tests for the diagnosis and treatment of malaria in southeast Nigeria: ex post and ex ante

**DOI:** 10.1186/1475-9276-9-1

**Published:** 2010-01-15

**Authors:** Benjamin SC Uzochukwu, Obinna E Onwujekwe, Nkoli P Uguru, Maduka D Ughasoro, Ogochukwu P Ezeoke

**Affiliations:** 1 Department of Community Medicine, College of Medicine, University of Nigeria, Enugu-campus, Nigeria; 2 Department of Health Administration and Management, College of Medicine, University of Nigeria, Enugu-campus, Nigeria; 3 Health Policy Research Group, College of Medicine, University of Nigeria, Enugu-campus, Nigeria; 4 Department of Paediatrics, University of Nigeria, Teaching Hospital Enugu, Nigeria

## Abstract

**Background:**

The introduction of rapid diagnostic tests (RDTs) has improved the diagnosis and treatment of malaria. However, any successful control of malaria will depend on socio-economic factors that influence its management in the community. Willingness to pay (WTP) is important because consumer responses to prices will influence utilization of services and revenues collected. Also the consumer's attitude can influence monetary valuation with respect to different conditions ex post and ex ante.

**Methods:**

WTP for RDT for Malaria was assessed by the contingent valuation method using a bidding game approach in rural and urban communities in southeast Nigeria. The ex post WTP was assessed at the health centers on 618 patients immediately following diagnosis of malaria with RDT and the ex ante WTP was assessed by household interviews on 1020 householders with a prior history of malaria.

**Results:**

For the ex ante WTP, 51% of the respondents in urban and 24.7% in rural areas were willing to pay for RDT. The mean WTP (235.49 naira) in urban is higher than WTP (182.05 Naira) in rural areas. For the ex post WTP, 89 and 90.7% of the respondents in urban and rural areas respectively were WTP. The mean WTP (372.30 naira) in urban is also higher than (296.28 naira) in rural areas. For the ex post scenario, the lower two Social Economic Status (SES) quartiles were more willing to pay and the mean WTP is higher than the higher two SES while in the ex ante scenario, the higher two SES quartiles were more WTP and with a higher WTP than the lower two SES quartile. Ex ante and ex post WTP were directly dependent on costs.

**Conclusion:**

The ex post WTP is higher than the ex ante WTP and both are greater than the current cost of RDTs. Urban dwellers were more willing to pay than the rural dwellers. The mean WTP should be considered when designing suitable financial strategies for making RDTs available to communities.

## Background

Malaria is highly endemic in Nigeria and it remains one of the major public health problem in Nigeria and a leading cause of morbidity and mortality in the country [1]. With a prevalence rate of 919/100,000, it accounts for 40% of disease burden reported at the public health facilities, 30% of all childhood deaths and 11% of maternal deaths. The loss to the economy as a direct result of malaria infections has been estimated as 132 billion Naira (US$1.01 billion at an exchange rate of 130 Naira = US$1.00) [2].

In most endemic countries, the diagnosis and treatment of malaria has traditionally relied on the clinical presentation of malaria symptoms due to lack of reliable microscopy in the majority of peripheral health units [3,4] and microscopical examination of Giemsa-stained blood films. Syndromic approach is unreliable because the symptoms of malaria are non-specific, overlapping with other febrile diseases. Studies in Africa have shown that more than 50% of patients clinically diagnosed with malaria have illnesses attributable to some other causes [5-7]. This results in over-diagnosis of malaria [8] over-prescription of antimalarial drugs, under-diagnosis and inappropriate treatment of non-malarial febrile illnesses (NMFI) [9-13].

On the other hand, even for the expert microscopist, microscopic diagnosis of malaria is time-consuming and labour-intensive. Although microscopy is considered to be the gold standard for malaria diagnosis [14,15] in many malaria-endemic areas like Nigeria, there is lack of trained microscopists and reliable equipment.

Rapid diagnostic tests have considerable potential as tool to improve the diagnosis of malaria [16,17]. And its use in malaria diagnosis is increasing in many countries as the result of their ease of use with minimal training [18]. Using RDTs for diagnosis at the community level will shorten the time between onset of symptoms and initiation of appropriate treatment, leading to less suffering by the infected individual and less potential to spread the parasite to others when the infected individual is bitten by more anopheline mosquitoes. RDT use should also help slow development of resistance and reduce costs by avoiding unnecessary use of antimalarials. And with the introduction of the costly artemisinin-based combination therapy (ACT) in sub-Saharan Africa, the use of RDTs has become more important in the control of malaria.

However any successful control of malaria will depend on socio-economic factors that influence its management in the community. There are multiple barriers to accessing formal healthcare among which is the cost of treatment [19,20].

The availability of increased funding for malaria through the Global Fund for the AIDS, Tuberculosis and Malaria will increase the ability of health services in many endemic countries to purchase RDTs in large numbers. A challenge these countries will face is the building of a long term financial sustainable mechanism that can continue to support RDT use if the availability of funds is reduced in the future as access and affordability will be critical for the utilization of RDT. Thus an important issue is how much people are willing and able to pay for it. Several facts influence this decision;- chance of utilizing such service, socio-economic status, availability of close alternative, prior exposure to product in question etc Furthermore, to make RDT easily accessible to all, government might need to take a decision on subsidy exemption and to do this, they require information on WTP.

In making pricing decisions, health program managers face an equity dilemma - the problem of balancing the need for programme sustainability with the social goal of making services available to low income clients [21] and hence increase coverage. Inferring from the law of demand it is generally difficult to achieve both coverage and sustainability especially in resource poor setting because as prices go up, demand will come down. Therefore willingness to pay (WTP) surveys allow programme managers to simulate price-related changes in demand without actually changing prices, giving them a way to make decisions based on empirical information. Thus, it also measures the potential demand for products or services by asking consumers, "Would you purchase this product if it were offered at this price?" They are frequently used in health, social and environmental programs for price setting and cost benefit analyses. WTP is important because consumer responses to prices will influence utilization of services and revenues collected. The consumer's attitude can also influence monetary valuation with respect to different conditions ex post and ex ante.

The correct population that has to provide their preferences in order to take resource allocation decisions in health care has been the subject of debate in the literatures [22]. There are theoretical arguments in favor of using members of the general population [23] as the relevant population and one reason is that when deciding whether a new health programme will be funded or not we should include the preferences of all subjects that can benefit from the program in the future. However, the main argument most frequently quoted in favor of using patients as subjects is that they are in a better position to value a medicine that improves their health. It has been argued that this framework provides a conservative estimate on WTP for health gains [24].

Individual preferences may not be independent of the consumer's vantage point. Allocations which appear desirable ex-ante may therefore not be so ex-post, since those who have used the product (ex-post) have a better understanding as there is no more health worker asymmetry of information. They are therefore more convinced in their decision be it price or making demand than those that have not be been exposed to the product (ex- ante). Thus differences observed between these groups represents to a greater extent the actual value of the product. In essence, the ex post WTP focuses on currently symptomatic persons who received the goods in question while the ex ante WTP focuses on currently non-symptomatic persons who are at future risk.

WTP has been used extensively in Nigeria for a variety of health care goods and services [25-28]. However, we are not aware of any WTP study in Nigeria with respect to RDTs. In this instance the WTP study sets out to identify the true demand for affordable and socially acceptable services, thus it is usually important to actively involve the consumers of these services in not only deciding if they want and can afford the test, but also if it satisfies their perceptions towards accurate diagnosis and cure of malaria [29].

The study therefore aims to determine WTP for RDTs across different socioeconomic groups in both urban and rural Nigeria where it has been newly introduced for the diagnosis of malaria in an era of change of first line antimalaria treatment to the expensive ACT. Since "WTP is inevitably associated with ability to pay" [30], the examination of WTP across socioeconomic and geographic groups could be used to ensure fairness in resource allocation. Furthermore to enact a policy, a reform has to be agreed, ex-ante preferred and ex-post desirable. Thus a reform which passes both tests is more likely to be enacted and has higher tendency to persist than one which does not. Since it is not possible to say anything about ex-post on the basis of ex-ante judgment, providing both ex-ante and ex-post information of willingness to pay for RDT to decision makers will be worthwhile.

## Methods

The study was undertaken in Enugu East Local Government Area (LGA) in Enugu State, South-east Nigeria. It has six districts (Abakpa-Nike, Mbulujodo, Mbulu Oweghe, Mbulu Iyiukwu, Emene and Trans Ekulu) with a 2006 population of 279,089 [31]. While Abakpa-Nike, Trans-Ekulu and Emene are urban districts, the rest are rural. It has 12 Public Health centres and 30 Private clinics and hospitals. The number of health workers in these health centres range from 3 to 15 with the highly developed ones having more workers than the rest of the centres. All the centres have drug dispensing units but no laboratory facilities. Malaria is holoendemic in the rural areas and meso-endemic in the urban areas

### Study Design

The study was part of a larger study that lasted for 30 months (to take care of the seasonal variations in malaria episodes) from 2005 to 2008 during which data was collected on all the different components of the study throughout the study period.

### Sample size calculation and sample selection

For the ex ante, the software for population survey in EPI Info 6 as used for sample size calculation. The parameters that were used for sample size calculation were a power of 80%, 95% confidence level and considering 2% as the proportion of people with malaria that used services from the least commonly visited providers (community health workers) [32] for first treatment of malaria and an average of 6% monthly malaria incidence rate in the state. Hence, using the minimum projected population of each rural district at 30,000 people and each urban area at 60,000 people, it was estimated that a minimum of 1800 and 3600 people will have malaria monthly in each rural and urban area. The calculated minimum sample sizes for each urban site is 240 and for each rural site 221. However, allowing for a 10% refusal rate and ensuring an adequate sample size for data analysis for each district, the estimated sample sizes are 250 for rural, giving a total sample size in the two rural areas of 500 and 270 for urban giving a total sample size in the two urban areas of 540. The overall sample size is 1020.

The Primary Health Care (PHC) house numbering system was used as the sampling frame to select at least 1020 households from the study areas using simple random sampling technique. The primary woman household caregiver or in her absence, her spouse were interviewed using questionnaire administered by trained interviewers. The reliability of the observations was ensured by thoroughly training the field workers, strictly supervising the field-work and pre-testing the study tools to ensure that they are content valid. Eventually, information was collected from 1131 respondents.

A sample size of 136 was computed as the required number of blood smear positive patients for the health center survey, where RDT was administered. The calculation of the sample size was based on the following parameters: expected sensitivity of a test = 90%, precision = 5% and alpha error = 0.05. This number (n = 136) was doubled taking into account a stratified analysis by age group category (0-4 years and 5 years and above). Similar parameters (with expected specificity of 90%) were used to calculate the required number of blood smear negative patients. The minimal final sample size was therefore fixed at 300 positive and 300 negative subjects, that is, 600 individuals for the RDT examination and therefore for the WTP ex post. All patients presenting at the centers with history of fever or non-specific symptoms or signs suggestive of malaria (e.g. headache only, chills, rigors) were consecutively recruited for the study.

### Data collection

After informed consent has been signed, using pre-tested interviewer-administered questionnaire, the researchers collected information from the patients or the care takers regarding their socio-economic and demographic background, and their willingness to pay for RDT. WTP for RDT for Malaria was assessed by the Contingent valuation method (CVM), a survey-based method to determine individuals' monetary valuation of health care or health states [33-35] using a bidding game approach in rural and urban communities in southeast Nigeria. The ex post WTP was assessed at the health centers on 638 patients immediately following diagnosis of malaria with RDT and the ex ante WTP was assessed by household interviews on 1131 householders with a prior history of malaria. The scenario that described the RDT was presented to the respondents before WTP was elicited. RDT was described as *a *new test kit which enables malaria to be diagnosed quickly even in remote areas, is easy to use by the health worker and the result is known within 15 minutes. In addition, for the ex post WTP, the respondents utilized the RDT. The starting amount used in the bidding game was derived from interviews conducted with Pharmacists to obtain the cost of RDTs in Enugu, Nigeria. Thus the market price was 200 Naira and we decided to mark up by 100 Naira for the starting bids and then increase or decrease by 100 Naira depending on the response to the starting bids. The respondents were allowed only three bids in order to decrease the complexity of the exercise to the respondents and the last bid represented their maximum WTP.

### Data analysis

The data was entered, prepared and analyzed using the statistical computer programme SPSS 15 for Windows and STATA. The economic level of the households was used as a local relative indicator of wealth among the included households. The Principal components analysis (PCA) in STATA software package was used to generate continuous socio-economic status (SES) index using information of the households' asset holdings together with the average weekly cost of food. The assets were key ones that included ownership of motorcar, motorcycle, radio, refrigerator, television set, fan and bicycle. The SES index was decomposed into quartiles (4 groups) of SES groups. The 4 SES groups were: the highest SES group (Q4) or least poor; SES group (Q3) or poor; SES group (Q2) or very poor and SES (Q1) or most poor. This was the used to measure the relationship between SES and WTP.

### Ethical considerations

Approval of the study was granted by the ethics committee of the University of Nigeria Teaching Hospital Enugu. Informed written consent was sought from the patients or parents/caretakers of underage patients.

## Results

### Socio-demographic characteristics of respondents

As shown in table 1, of the 638 patients recruited for this study, 317 (49.7%) and 321 (50.3%) were from the urban and rural areas respectively. Majority of the respondents 423(66.3%) were above 30 years and more than a third of the ill persons 244 (38.2%) were below 5 years of age. A majority of the ill persons 439 (68.8%) are females, 538 (84.3%). Most of them (81.7%) had one form of formal education or the other. Their occupational status shows that petty traders are 139(21.8%), farmers 129(20.2), civil servants 97(15.2), self employed 101(15.8%) and 91 (14.3%) are unemployed.

**Table 1:** Respondents' socio-economic and demographic characteristics (Health center survey)

	Urban N = 317	Rural N = 321	Combined N = 638
Age of respondent (years): Mean (SD)	33.1 (9.7)	35.8 (12.7)	34.4 (11.4)

Age of ill person: Mean (SD)	8.3 (8.7)	17.6 (17.8)	13.0 (14.8)

Sex of respondents			
Females: n %	287 (90.5)	280 (87.2)	567 (88.9)
Males: n %	30 (9.5)	41 (12.8)	71 (11.1)

Sex of ill person			
Females: n %	215 (67.8)	224 (69.8)	439 (68.8)
Males: n %	102 (32.2)	97 (30.2)	199 (31.2)

Years of education: Mean (SD)	9.2 (4.5)	6.7 (5.0)	

Whether married: n %	257 (81.1)	281 (87.5)	538 (84.3)

Occupation: n %			
Farmer	19 (6.0)	110 (34.3)	129 (20.2)
Unemployed	58 (18.3)	33 (10.3)	91 (14.3)
Civil servant	99 (31.2)	40 (12.5)	97 (15.2)
Petty trading	45 (14.2)	52 (16.2)	139 (21.8)
Self employed	1 (0.3)	3 (0.9)	101 (15.8)
Big business man/woman	17 (5.4)	2 (0.6)	19 (3.0)
Private employment	28 (8.8)	13 (22.7)	4 (0.6)
Others	27 (8.5)	3 (0.9)	30 (4.7)
Total	317 (100)	321 (100)	638 (100)

SES quartiles			
Q1 (most poor)	61 (19.2)	99(30.8)	160 (25.1)
Q2 (very poor)	46 (14.3)	114 (35.5)	160 (25.1)
Q3 (poor)	97 (30.6)	62 (19.3)	159 (24.9)
Q4 (least poor)	113 (35.6)	46 (14.3)	159 (24.9)
Total	317 (100)	321 (10)	638 (100)

In the household survey, the numbers of complete questionnaires that were available for data analysis were 576 and 555 in Urban and rural areas respectively (Table 2). The table shows that the respondents were mostly females in both areas. The respondents from the urban areas were expectedly more educated than those from the rural areas. The average years of formal schooling were 10.7 years in Urban and 7.1 years in rural area respectively. Most of the respondents in both areas were middle-aged, while the mean number of persons in a household is 5.5 and 4.7 in urban and rural areas respectively.

**Table 2:** Household Respondents' socio-economic and demographic characteristics

	Urban N = 576	Rural N = 555	Combined N = 1131
Respondent' status			
Female head of household: n %	22 (3.8)	119 (21.4)	**141 (12.5)**
Male head of household: n %	16 (2.8)	48 (8.6)	**64 (5.7)**
Wife: n %	442 (76.7)	349 (62.9)	**791 (69.9)**
Grandmother: n %	13 (2.2)	6 (1.1)	**19 (1.7)**
Representative of household: n %	83 (14.4)	33 (5.9)	**116 (10.3)**

Whether married: n %	498 (86.5)	498 (89.7)	996 (88.1)

People in the household: Mean (SD):	5.5 (3.9)	4.7 (2.4)	**5.2 (3.3)**

Age of respondent (years): Mean (SD)	36.4 (11.3)	40.2 (14.5)	38.2 (13.1)

Sex of respondents			
Females: n %	541 (93.9)	515 (92.8)	1056 (93.4)
Males: n %	35 (6.1)	40 (7.2)	75 (6.6)

Years of education: Mean (SD)	10.7 (3.7)	7.1 (5.7)	8.9 (5.1)

Occupation: n %			
Farmer	21 (3.6)	190 (34.2)	211 (18.7)
Unemployed	86 (14.9)	66 (11.9)	152 (13.4)
Petty trading	261 (45.3)	103 (18.6)	364 (32.2)
Civil servant	96 (16.7)	68 (12.3)	164 (14.5)
Private employment	20 (3.5)	19 (3.4)	39 (3.4)
Big business man/woman	19 (3.3)	36 (6.5)	55 (4.9)
Self employed	44 (7.6)	59 (10.6)	103 (9.1)
Others	15 (2.6)	20 (3.6)	35 (3.1)

SES quartiles			
Q1 (most poor): n %	222 (38.5)	61 (11.0)	283 (25)
Q2 (very poor): n %	78 (13.5)	204 (36.8)	282 (24.9)
Q3 (poor): n %	152 (26.4)	131 (23.6)	283 (25.0)
Q4 Least poor): n %	124 (21.6)	159 (28.6)	283 (25.0)

### Willingness to Pay

As shown in table 3, for the ex post WTP, the willingness to pay for RDTs was positive for a majority of the respondents in both rural (90.7%) and urban (89%). The mean WTP in urban (372.30 naira) is higher than in rural areas (296.28 naira). In the combined data, the willingness to pay for RDTs was positive in 89.8% of the respondents and the mean WTP was 335.1 Naira. There was no statistical significant difference in WTP for RDT between the urban and rural dwellers (p > 0.05). For the ex ante WTP, the willingness to pay for RDTs was positive in 51% of the respondents in urban and 24.7% in rural areas. The mean WTP in urban (235.49 naira) is higher than mean WTP in rural areas (182.05 Naira). In the combined data, the willingness to pay for RDTs was positive in 38.1% of the respondents and the mean WTP was 209.27 Naira and there was a statistically significant difference in WTP for RDT between the urban and rural dwellers (p < 0.05). In general, the ex post WTP was higher than the ex ante WTP.

**Table 3:** Willingness to pay for RDT (130 Naira = 1 US Dollars)

Ex Post WTP	Urban N = 317	Rural N = 321	Combined N = 638	Chi square (p value) Urban/Rural
WTP: n (%)	282 (89.0)	291 (90.7)	573 (89.8)	0.5 (0.479)
Mean (SD) WTP (Naira)	372.3 (205.14)	296.3 (188.72)	335.1 (199.8)	NA

				

**Ex ante WTP**	**Urban** **N = 576**	**Rural** **N= 555**	**Combined** **N = 1131**	**Chi square (p value)** **Urban/Rural**

WTP: n (%)	294 (51.0)	137 (24.7)	431 (38.1)	83.25 (0.0001)*
Mean (SD) WTP (Naira)	235.49 (189.8)	182.05 (162.02)	209.27 (178.6)	NA

NA = Not applicable

*Statistically significant

Ex ante WTP was directly dependent on cost with 68.2%, 38.1% and 16.5% of respondents indicating willingness to pay 200 Naira, 300 Naira and 400 Naira respectively. Also, the ex post WTP was directly dependent on cost with 69.3%, 55.7% and 20.2% of respondents indicating willingness to pay 200 Naira, 300 Naira and 400 Naira respectively (table 4).

**Table 4:** Respondents' willingness to pay amount

WTP amount (naira)	Ex post WTP N = 573 N (%)	Ex ante WTP N = 431 N (%)
200.00 and less	397 (69.3)	294 (68.2)
300.00	319 (55.7)	164 (38.1)
400.00 and more	116 (20.2)	71 (16.5)

### Socioeconomic status (SES) differences in Willingness to Pay

In the ex post WTP, the willingness to pay for RDTs was positive in 92.5 and 91.9% of the respondents in the lower SES quartiles (Q1 and Q2) respectively which was more than the 88.1 and 86.8% of the respondents in the higher SES (Q3 and Q4) respectively that recorded a willingness to pay. There was no statistically significant differences between them (p > 0.05). The mean WTP (360.5 and 346.6 Naira) for Q1 and Q2 respectively are higher than in Q3 and Q4 which are 294.4 and 338.7 Naira respectively (Table 5).

**Table 5:** Socioeconomic status (SES) differences in WTP for RDT. (130 Naira = 1 US Dollars)

	Ex post WTP N = 573		Ex ante WTP N = 431	
**SES quartiles**	**WTP** **No (%)**	**Mean (SD) WTP** **(Naira)**	**WTP** **No (%)**	**Mean (SD) WTP** **(Naira)**

Q1 (most poor):	148 (92.5)	360.5 (240.3)	79 (27.9)	158.90 (146.2)
Q2 (very poor)	147 (91.9)	346.6 (215.3)	86 (30.5)	194.79 (175.9)
Q3 (very poor):	140 (88.1)	294.4 (136.5)	133 (47.0)	232.54 (162.2)
Q4 (Least poor):	138 (86.8)	338.7 (201.2)	133 (47.2)	251.67 (210.4)
Total:	573 (89.8)	335.1 (199.8)	431 (38.1)	209.27 (178.6)
Chi square for trend	4.13	NA	38.7	NA
P value	0.248	NA	0.000*	NA

NA = Not applicable

*Statistically significant

For the ex ante WTP, the willingness to pay was positive for 47.0 and 47.2% of the respondents in the higher SES quartiles (Q3 and Q4) respectively which was more than the 27.9 and 30.5% of the respondents in the lower SES (Q1 and Q2) respectively that recorded willingness to pay. The difference was statistically significant (p < 0.05). The mean WTP (158.9 and 194.79 Naira) for Q1 and Q2 respectively are lower than in Q3 and Q4 which are 232.54 and 251.67 Naira respectively (Table 5).

## Discussion

In this study the currently symptomatic person (those who visited the health centers for RDT diagnosis) has more willingness to pay for the RDT. This is similar to what was obtained in Myanmar where those who were symptomatic had more willingness to pay for the diagnostic technology [36]. In microeconomic theory, demand-determining variables included tastes of the commodity consumed, among others[37]. In our study and in the Myanmar study [36], it was considered that the respondents provided the answer in accordance with their current taste of the RDT and thus better appreciates its benefits. Thus the decision to pay for RDT was propelled by need. It has also been noted that a recent episode in a household was associated with increased WTP [38].

In the ex ante scenario, the urban dwellers were more willing to pay for RDT. This might be due to the fact that they are more educated and therefore appreciate the usefulness of RDT. The mean years of education in urban is 10.7 years as against 7.1 years for the rural dwellers. The level of education has been significantly linked to WTP [25,27] because with increasing education level, there is assumed higher awareness about health care needs, resulting in the will to pay for personal and household protection [25].

The mean WTP of the poorest quartiles for the ex ante scenario was less than those from the least poor quartiles. It has been noted that the more income an individual has, the greater the WTP, which is consistent with welfare economic theory [36]. However, the response is different with those who utilized RDT. Here the poorest were more willing to pay and to even pay a higher amount. And this is contrary to the findings of Donaldson et al. [30] that showed that those with a lower ability to pay were less likely to be willing to pay for healthcare. The reason for the poorest being more willing to pay might be because they are poor and therefore suffer more from malaria and their experience with the use of RDT might be seen by them as a way of treating the malaria faster and thus saving them some cost. It has been noted that socioeconomic differentials exists in access to malaria interventions increasing the vulnerability of the poorest [39]. Despite the fact that the poor are willing to pay for RDTs, it is important to ensure that strategies put in place to improve the management of malaria such as RDTs are equitable and available and accessible to the poorest SES groups because they bear a disproportionate burden of the disease and have poor health-seeking behavior [40].

The fact that the elicited WTP ex post and ex ante are greater than the current market price of RDT is quite encouraging and is a reflection of the fact that the community value RDTs highly and are willing to utilize it if available. In some contexts charging for malaria prevention strategies like mosquito nets was associated with high levels of uptake among the poorest quintiles [38]. Thus the government and healthcare providers can be assured of sustained financial mechanisms to ensure constant availability and increased coverage.

It should be noted that on the whole, the use of RDTs lowers the cost of malaria because they produce quick and accurate diagnosis of malaria, thereby decreasing the length of morbidity, while also increasing productivity. It is therefore worthwhile encouraging the communities to utilize it. Within the context of increasing antimalarial costs and or decreasing malaria transmission, the importance of limiting antimalarial treatment to only those confirmed as having malaria parasites is very important [41]. Therefore the use of RDTs would considerably reduce over-treatment and wrong diagnosis which is seen in cases where presumptive diagnosis is used [42] thereby reducing the cost of treating malaria to the individual.

Although the respondents were asked how much they were willing to pay for RDTs after narrating the scenario to them and after diagnosis with RDTs as the case may be, we did not ask the respondents to state why they would like to pay more or less than the market price of RDT. It is our believe that their responses is a reflection of their valuation of the RDTs. However, we note this as a limitation of the study and a gap that needs to be filled in future studies with more appropriate qualitative methods.

## Conclusions

Respondents are willing to pay for RDTs and ex post WTP is higher than the ex ante WTP. For a wider distribution of RDTs in Nigeria to enable fast diagnosis and correct treatment of malaria cases with ACT, the government should consider providing the RDTs free of charge or subsidize them for the poorest socio-economic status consumers (despite their willingness to pay) and other vulnerable groups, like pregnant women. This is important because setting the user fees at mean WTP for instance does not necessarily ensure universal access as all those with below mean WTP will be denied access while those with above mean WTP will earn consumer surplus. Therefore, government and donor agencies should consider the possibility for subsidization - public or private - and/or the possibility of price discrimination. The National Insurance Scheme (NHIS) could provide this cross subsidization if the NHIS is scaled up in Nigeria.

Furthermore, government and donors should appreciate the benefits of decreasing overall societal costs of malaria and increasing societal productivity if RDTs are appropriately deployed for diagnosis and subsequent treatment of malaria, and this calls for public financing of the intervention. However, social marketing and health education campaigns can be used to convince householders on how to convert some of their current expenditures on some inappropriate treatment to purchase of RDTs for diagnosing malaria. Governments and donors should be willing to commit funds to make RDTs affordable especially to the poorest consumers and other vulnerable members of the society if the intervention is to be used to significantly reduce the burden of malaria. We are aware that this single study may not be a guarantee for generalization in Nigeria, but it provides some evidence to guide policy makers.

## Competing interests

The authors declare that they have no competing interests.

## Authors' contributions

BSCU conceived and designed the study, BSCU, MDU collected the data, BSCU and OEOanalysed the data. BSCU wrote up the manuscript with input from all the authors. All authors read and approved the final manuscript.
